# Cytogenetic Analysis of Altitude‐Associated Chromosome Variations in *Campeiostachys nutans* From Western Sichuan Plateau Using Multicolor GISH and ND‐FISH Approaches

**DOI:** 10.1002/ece3.73904

**Published:** 2026-06-30

**Authors:** Cairong Yang, Jiezhi Yang, Yi Ou, Yueju Zhou, Zhimeng Wang, Weiliang Qi, Dingfang Luo, Yutong Qiao

**Affiliations:** ^1^ College of Chemistry and Life Science Chengdu Normal University Chengdu Sichuan China; ^2^ Sichuan Provincial Forestry and Grassland Key Laboratory of Biodiversity Conservation and Sustainable Community Development in Giant Panda National Park Chengdu Sichuan China; ^3^ Neijiang Academy of Agricultural Sciences Neijiang Sichuan China; ^4^ College of Agriculture and Forestry Longdong University Qingyang Gansu China

**Keywords:** (AAG)_10_, (GAA)_7_, 5S rDNA, *Campeiostachys nutans*, genetic diversity, ND‐FISH, pAs1

## Abstract

*C. nutans* , a highly advantageous forage grass on the Western Sichuan Plateau, remains poorly studied in terms of its chromosomal structural diversity in this region. The genomic and chromosomal organization of *C. nutans* was investigated using GISH and ND‐FISH with four oligonucleotide probes. The results showed that repetitive sequence distribution exhibited genome‐specific patterns: the H genome demonstrated high structural stability with low polymorphism, while the St genome showed moderate variation, and the Y genome displayed the highest polymorphism, with chromosomes 1Y, 2Y, and 7Y identified as variation hotspots. Signal density and polymorphism varied with altitude; the materials from middle and low altitudes exhibited relatively higher genetic polymorphism. Notably, (AAG)_10_ and (GAA)_7_ probes revealed altitudinal trends, with signal abundance peaking at mid‐elevations before declining at higher altitudes. 5S rDNA and pAs1 distributions further highlighted conserved loci alongside altitude‐dependent polymorphisms. Karyotypic analysis based on probe combinations confirmed uneven intragenomic variation, with 3H, 1St/3St, and 1Y/7Y showing the highest diversity. These findings underscore the differential genome plasticity in *C. nutans* , with the Y genome being the most dynamic, possibly as an adaptive response to environmental gradients. The study provides insights into the evolutionary mechanisms and altitude‐driven genomic diversification in polyploid grasses.

## Graphical abstract

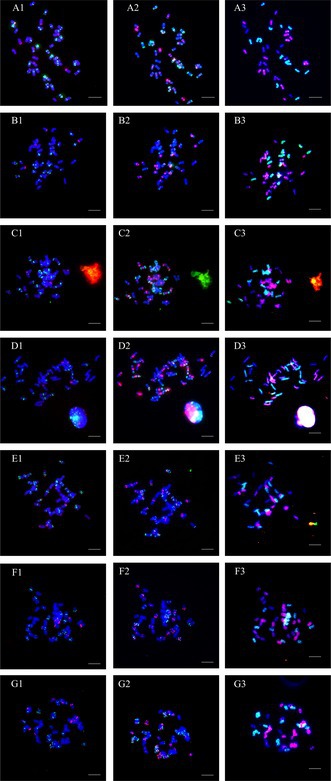

## 1 Introduction

The Qinghai‐Tibet Plateau, as known as the third pole of the Earth because of its high altitude, provides favorable conditions for the growth and development of many plants. The Qinghai‐Tibet Plateau is also a hot area to study plant genetic diversity and plant environmental adaptation mechanism. *Campeiostachys nutans* is an important forage grass in *Campeiostachys* (Triticeae, Poaceae), widely distributed in Inner Mongolia, Sichuan, Xizang and Xinjiang provinces, China. The Western Sichuan Plateau, located at the junction of the Qinghai‐Tibet Plateau and the Hengduan Mountains, is characterized by high elevation, low temperature and harsh environments, and represents one of the global biodiversity hotspots in China (Zhang et al. 2016). As the largest highland grassland in Sichuan, *C. nutans* is the most advantageous forage in Western Sichuan Plateau. *C. nutans* is a hexaploid with StYH genomic constitution (Lu 1993). *C. nutans* was first discovered and named *Elymus nutans* by the German botanist Grisebach (Grisebach 1868). *Elymus* L. s. l., circumscribed by Löve (1984) and Dewey (1984), constitutes the largest genus of Triticeae, comprising around 150 worldwide‐distributed polyploid species that harbor diverse combinations of five fundamental genomes: St, H, Y, P and W. *Elymus nutans* has recently been reclassified into the genus *Campeiostachys* based on its StYH genome composition (Yen and Yang 2011). Zheduo Mountain, a major pass situated on the eastern margin of the Qinghai‐Tibet Plateau within the Hengduan Mountain range, serves as a prominent geographical and ecological transition zone. Its notable altitudinal gradient‐ranging from approximately 2600 m to over 4600 m‐fosters diverse ecosystems and makes the mountain an ideal natural laboratory for scientific research. This site is particularly valuable for studies in ecology, botany, and genetics, offering insights into high‐altitude adaptation mechanisms in plant species such as *C. nutans* .

The genetic diversity of *C. nutans* populations from Qinghai‐Tibet Plateau has been investigated by molecular marker tool or cytogenetic methods. It was found that there was relatively high within and among‐geographical group diversity wby using ISSR markers. Research also revealed significant geographical differences of *C. nutans* between the Qinghai‐Tibet Plateau and Xinjiang (Chen et al. 2009). Cytogenetic analyses have further elucidated chromosomal‐level variation in *C. nutans* . In 2011, FISH combined with Gish were used to study the chromosomal structure of *C. nutans* (Dou et al. 2011). High FISH pattern polymorphism and chromosome variation were detected within and among populations of *C. nutans* (Dou et al. 2017). Subsequent studies demonstrated that chromosomal abnormalities during meiosis occur at varying frequencies across subgenomes and individual chromosomes in heterozygous plants of *C. nutans* (Liu et al. 2022a). Notably, Liu et al. (2022b) observed an uneven distribution of chromosomal variants between genomes and homologous chromosomes, with reduced‐fertility plants exhibiting greater chromosomal variation than normal‐fertility specimens. Recent advances include the identification of five species‐specific chromosomal rearrangement (CRs) in the St, Y, and H genomes using fifty‐nine single‐gene FISH probes (Liu et al. 2023), and the discovery of four distinct translocation types among subgenomes in wild populations from the Qinghai‐Tibet Plateau (Liu et al. 2024). The latter study also reported intra‐ and inter‐population variation in the distribution and abundance of S5 and AAG FISH signals. Complementary work by Li et al. (2024) revealed substantial genetic diversity in *C. nutans* populations at both nuclear and plastid DNA levels, with cpDNA sequences and microsatellite markers demonstrating that most variation occurs within the populations. Although some studies have been conducted on the genetic diversity of *C. nutans* on the Qinghai‐Tibet Plateau, research on the structural genetic diversity of chromosomes in *C. nutans* from the Western Sichuan Plateau remains limited.

Polyploidy, defined as the presence of three or more complete chromosome sets within a nucleus, serves as a fundamental mechanism in plant evolution and genome diversification (Otto and Whitton 2000). This phenomenon is particularly significant for environmental adaptation, with numerous studies demonstrating that polyploid plants exhibit enhanced stress tolerance and greater capacity to colonize extreme or marginal habitats (te Beest et al. 2012; Ramsey and Ramsey 2014; Van de Peer et al. 2021). The ecological implications of polyploidy are multifaceted. By interfering with morphology and physiology, genome size may be associated with ecological preferences in the following areas: alternative variables for latitude and altitude, or potential bioclimatic variables and habits ultimately affect adaptability diversification (Souza et al. 2019; Carta and Peruzzi 2016; Trávníček et al. 2019; Faizullah et al. 2021). These adaptations are particularly evident in regions with high polyploid frequencies, such as the Qinghai‐Tibet Plateau and northwestern China (Wang, Zhou, et al. 2023; Wang, Deng, et al. 2023). Chromosome number and genome size, partially correlated with bioclimatic variables and altitude, are formed through neutral evolution and adaptive evolution in Maxillariinae (Moraes et al. 2022). Environmental factors exert strong selective pressures on polyploid evolution. The autotetraploidization event of citrus is related to the genetic composition of genotypes and influenced by the plant growth environment (Guerra et al. 2016). Cold temperatures have been identified as a potential driver of polyploidization (Glennon et al. 2024), while specific case studies reveal differential responses to climate change between ploidy levels. For instance, tetraploid Neobatrachus demonstrates greater resilience to habitat loss compared to its diploid counterparts, likely due to enhanced gene flow (Novikova et al. 2020). Similarly, allopolyploid wheat benefits from LHP1‐mediated epigenetic regulation, which confers genomic plasticity and improved stress responses (Li et al. 2023). Physiological advantages are further exemplified in *Dianthus broteri*, where higher ploidy levels (12×) show superior heat tolerance through maintained photosynthetic efficiency and water balance (López‐Jurado et al. 2024). At the genomic level, chromosomal rearrangements, particularly translocations and inversions, play crucial roles in polyploid evolution and speciation (Rieseberg 2001; Faria and Navarro 2010). These structural variations, including species‐specific CRs, are thought to play an important role in genome evolution and speciation in polyploid plants (Gill 1991). Environmental influences on chromosome recombination have been well‐documented in several genera, including *Triticum*, *Kengyilia*, and *Elymus* (Badaeva et al. 1995; Wang et al. 2012; Yang et al. 2017). The complex interplay between polyploid evolution and environmental factors underscores the need for continued investigation into how genetic modifications facilitate adaptation to changing ecological conditions.

*C. nutans* is a dominant and endemic grass species on the Western Sichuan Plateau. It serves as a crucial forage resource for alpine pastoral ecosystems and a valuable gene pool for the genetic improvement of Triticeae crops, with important ecological and agricultural significance. However, studies on its chromosomal genetic diversity in this region remain scarce. To provide evolutionary data for *C. nutans* from the perspective of chromosomal diversity, multicolor fluorescence in situ hybridization (FISH) and genomic in situ hybridization (GISH) were employed in the present study to identify chromosome structural variations in *C. nutans* populations. These populations were collected across an altitude gradient of 2600–4600 m on Zheduo Mountain, Sichuan Province, China. The relationships between chromosomal structural polymorphism and environmental factors were discussed.

## 2 Materials and Methods

### 2.1 Plant Material

The root tips of seven materials of *C. nutans* were used in this study. Three individuals were randomly selected at 10‐m intervals within each population as experimental materials. The material collection information is presented in Table 1. In this study, materials collected from elevations below 3000 m were defined as the low‐elevation group, those from 3000 to 4000 m as the medium‐elevation group, and those above 4000 m as the high‐elevation group. The seeds of *Pseudoroegneria libanotica* (2*n* = 2*x* = 14; StSt; St donor of *C. nutans* ) and *Hordeum bogdanii* (2*n* = 2*x* = 14; HH; H donor of *C. nutans* ) were kindly provided by National Plant Germplasm System (Pullman, WA, USA) and Triticeae Research Institute of Sichuan Agricultural University.

**TABLE 1:** Source of *C. nutans* samples.

Species	Code	Altitude (m)	Habitat	Source
*C. nutans*	ENU0402	2616.24	Rock slope	Zheduo Mountain, Kangding, Garzê, Sichuan, China
*C. nutans*	ENU0403	2939.92	Grass slope	Zheduo Mountain, Kangding, Garzê, Sichuan, China
*C. nutans*	ENU0404	3177.01	Rock slope	Zheduo Mountain, Kangding, Garzê, Sichuan, China
*C. nutans*	ENU0406	3478.48	Grass patches	Zheduo Mountain, Kangding, Garzê, Sichuan, China
*C. nutans*	ENU0408	3777.09	Grass slope	Zheduo Mountain, Kangding, Garzê, Sichuan, China
*C. nutans*	ENU0409	4114.64	Grass slope	Zheduo Mountain, Kangding, Garzê, Sichuan, China
*C. nutans*	ENU0416	4278.28	Grass slope	Zheduo Mountain, Kangding, Garzê, Sichuan, China

### 2.2 Chromosome Preparation

Seeds were germinated on moist filter paper in petri dishes at 23°C. Root tips with a length of 1 cm were collected and immediately transferred into 1.5 mL microcentrifuge tubes containing distilled water. The samples were subjected to cold pretreatment at 4°C in an ice‐water bath for 28 h to synchronize cell division. Following pretreatment, specimens were fixed in 90% glacial acetic acid for 10–15 min at room temperature, with subsequent triple rinses in distilled water to remove residual fixative.

To prepare the citrate buffer, accurately weigh 0.5707 g of trisodium citrate and 0.4324 g of citric acid into a suitable container. Subsequent to complete dissolution, adjust the final volume of the mixture to 50 mL using deionized water and vortex thoroughly to ensure homogeneity. For the fabrication of the 10 mL enzyme solution, precisely weigh 0.4 g of cellulase Onozuka R‐10 (Yakult Pharmaceuticel Ind. Co. Ltd., Japan) and 0.2 g of pectolyase Y‐23 (Kyowa Chemical Products Co. Ltd., Japan) into a clean vessel. Gradually add 10 mL of the aforementioned freshly prepared citrate buffer to the enzyme mixture, followed by gentle agitation until the enzymes are fully solubilized.

The meristematic regions (approximately 1–2 mm) were carefully dissected and enzymatically digested in 20 μL of digestion buffer containing 4% cellulase (w/v) and 2% pectinase (w/v) at 37°C for 50 min in a temperature‐controlled water bath. Post‐digestion, the enzyme solution was carefully removed and the samples were washed twice with 70% ethanol. The root tips were then gently homogenized in glacial acetic acid using fine dissection needles to release intact chromosomes, followed by centrifugation at 6000× *g* for 2 min at room temperature. The supernatant was discarded and the pellet was resuspended in fresh glacial acetic acid.

For slide preparation, 5 μL aliquots of chromosome suspension were dropped onto pre‐cleaned microscopic slides. Chromosome spreads were examined under phase‐contrast microscopy, and slides exhibiting optimal chromosome dispersion were selected and stored in a desiccator at room temperature for subsequent analyses. The duration of enzymatic digestion was empirically adjusted according to tissue condition and ambient temperature, ranging from 50 to 55 min.

### 2.3 Non‐Denaturing Fluorescence In Situ Hybridization (ND‐FISH)

Oligonucleotide probes (AAG)_10_, 5S rDNA, pAs1 and (GAA)_7_ were used as the probes in ND‐FISH. The pAs1 probe originates from non‐coding repetitive DNA of *Aegilops squarrosa* , the D‐genome progenitor of wheat, while the 5S rDNA probe cloned from common wheat genomic libraries binds specifically to the 5S rRNA gene array and is extensively exploited in Triticeae cytogenetics (Cuadrado et al. 1997; Sergeeva et al. 2017). The probes used in FISH were synthesized by Sangon Biotech Co. Ltd. (Shanghai). The (AAG)_10_ and 5S rDNA (Lang et al. 2019) probes were labeled with TAMRA (red) fluorescent dye at the 5′ end, while the pAs1 (Danilova et al. 2012) and (GAA)_7_ probes were modified with FAM (green) fluorescent dye at the 5′ end. The FISH procedure was performed according to the method described by Li and Yang (2022) with minor modifications. The hybridization mixture (10 μL per slide), consisting of 1 μL probe (10 μmol/mL), 4 μL 1 × TE buffer and 4 μL 2 × SSC buffer, was prepared on ice with adjustable probe concentrations based on signal intensity requirements. The mixture was applied to the target area of slides, covered with coverslips, and hybridized in a pre‐warmed humid chamber at 42°C for 2 h. ND‐FISH eliminated the need for high temperature denaturation. For post‐hybridization, slides were washed in 2 × SSC until coverslip detachment, rinsed with distilled water, air‐dried, and counterstained with 12 μL Antifade Mounting Medium with DAPI (Vector Laboratories Inc., USA) for 15 min in darkness. All procedures involving hybridization mixture preparation and staining were conducted under low‐temperature conditions with light protection throughout the process.

### 2.4 Genomic In Situ Hybridization (GISH)

Genomic DNAs of *P. libanotica* and *H. bogdanii* were extracted using the Rapid Plant Genomic DNA Isolation Kit (Sangon Biotech, Shanghai, China) and used as probes in GISH. The DNA of *Pse. libanotica* and *H. bogdanii* was probed using the Atto488 NT Labeling Kit and Atto550 NT Labeling Kit (Jena Bioscience, Germany), respectively. GISH was carried out following the procedure of Yang et al. (2017). Following two rounds of fluorescence in situ hybridization (FISH), the slides were regenerated by incubation in 70% ethanol preheated to 60°C for 5 min before proceeding to genomic in situ hybridization (GISH). For each slide, 10 μL of hybridization mixture was formulated by mixing 1 μL of each probe (140 ng/μL), 4 μL of 1 × TE buffer, and 4 μL of 2 × SSC buffer. GISH analysis was adapted from conventional non‐denaturing FISH protocols, incorporating pre‐hybridization denaturation (100°C, 2 min) and an overnight hybridization.

### 2.5 Microscopy and Image Processing

Chromosomal fluorescence signals were examined under oil‐immersion optics using a Leica (Germany) DM series microscope integrated with a DFC7000T digital imaging system. Subsequent image processing and analysis were performed using Adobe Photoshop CC 2024 imaging software with standardized parameters. For each individual, the chromosomal FISH results were observed and counted in at least three cells.

### 2.6 Statistics of Hybridization Signals

Each chromosome is divided into two distinct regions, the short arm (S) and the long arm (L), with the centromere serving as the reference point. Based on the relative physical position of the signals on the chromosome, the chromosome is further subdivided into the centromeric region, pericentromeric region, subtelomeric region, and telomeric region. A hybridization locus is defined as a clearly distinguishable punctate or diffuse fluorescent signal on the chromosome with intensity significantly higher than the background fluorescence. Signals at different positions within the same chromosome arm are recorded as separate loci. Weak signals, diffuse background signals, and non‐specific signals are excluded from statistical analysis.

A hybridization locus on homologous chromosomes is classified as a polymorphic locus if it meets any of the following criteria: (1) Presence–absence polymorphism of signals: In the same region of a pair of homologous chromosomes, a signal is detected on one chromosome but absent on the other. (2) Signal position polymorphism: Hybridization signals are present on both homologous chromosomes, but the chromosomal regions where the signals are located differ. (3) Signal number polymorphism: The number of hybridization signals at the same chromosomal locus varies significantly; for instance, one chromosome exhibits one signal spot, while the other shows two or more signal spots. (4) Signal intensity polymorphism: Obvious differences in signal intensity are observed, as confirmed by two independent observers. Loci that are stably present across all examined samples with consistent position and intensity are defined as conserved loci and are not included in the polymorphism statistics.

The mean ± standard deviation (SD) of probe signals was calculated in Microsoft Excel 2021. One‐way analysis of variance (ANOVA) was conducted, and significant differences among groups were evaluated by multiple comparison tests with SPSS 26.0. The correlation analysis of karyotype indicators versus altitude was conducted in R with ggplot2.

## 3 Results

### 3.1 Distribution of Repetitive Sequences in the H Genome of *C. nutans*

**FIGURE 1:**
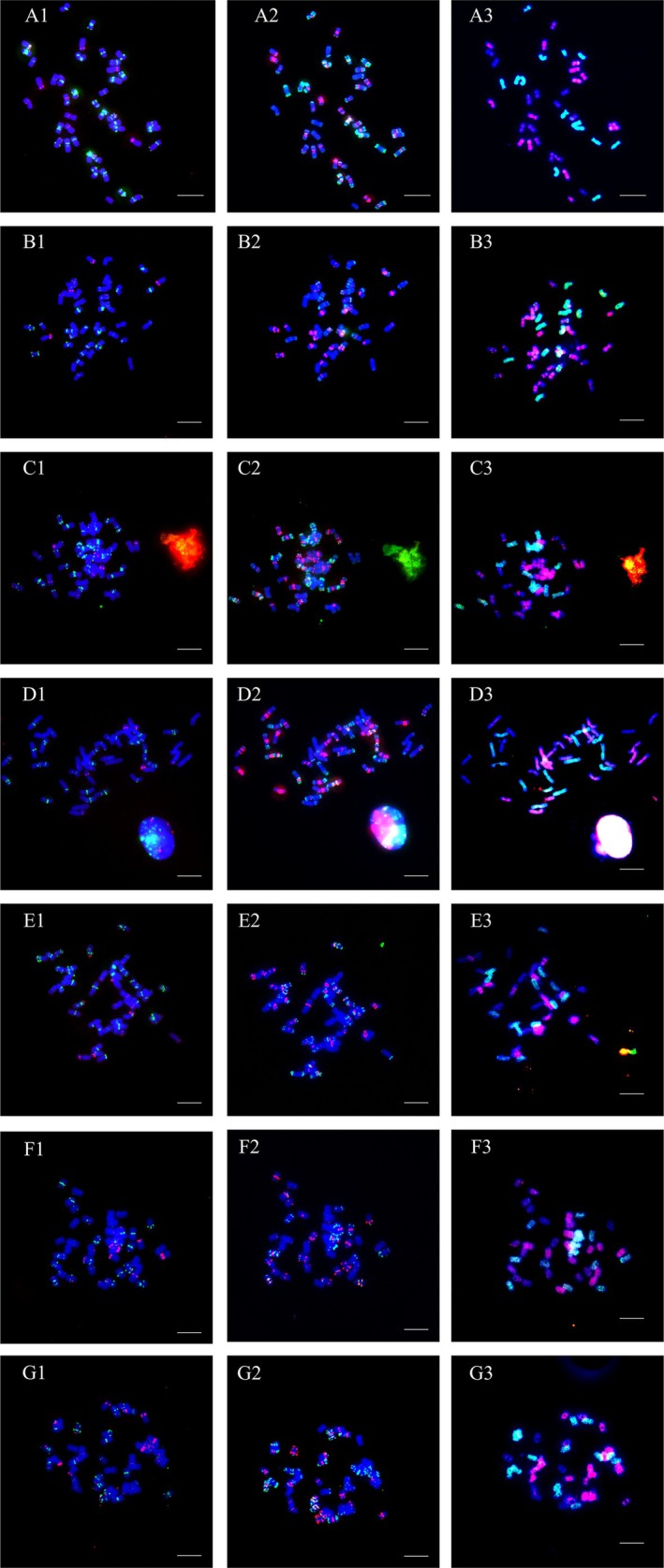
Distribution patterns of 5S rDNA and repetitive sequence probe combinations (GAA)_7_, (AAG)_10_, and pAs1 on chromosomes of *C. nutans* from seven different altitudes Annotation: A1, B1, C1, D1, E1, F1, G1: FISH hybridization signal patterns of 5S rDNA (red) and (GAA)_7_ (green) probes. A2, B2, C2, D2, E2, F2, G2: FISH hybridization signal patterns of (AAG)_10_ (red) and pAs1 (green) probes. A3, B3, C3, D3, E3, F3, G3: GISH hybridization signals using *P. libanotica* (red) and *H. bogdanii* (green) genomic DNA as the probe. A–G: Represent *C. nutans* samples ENU0402, ENU0403, ENU0404, ENU0406, ENU0408, ENU0409 and ENU0416, respectively. Scale bar = 10 μm.

**FIGURE 2:**
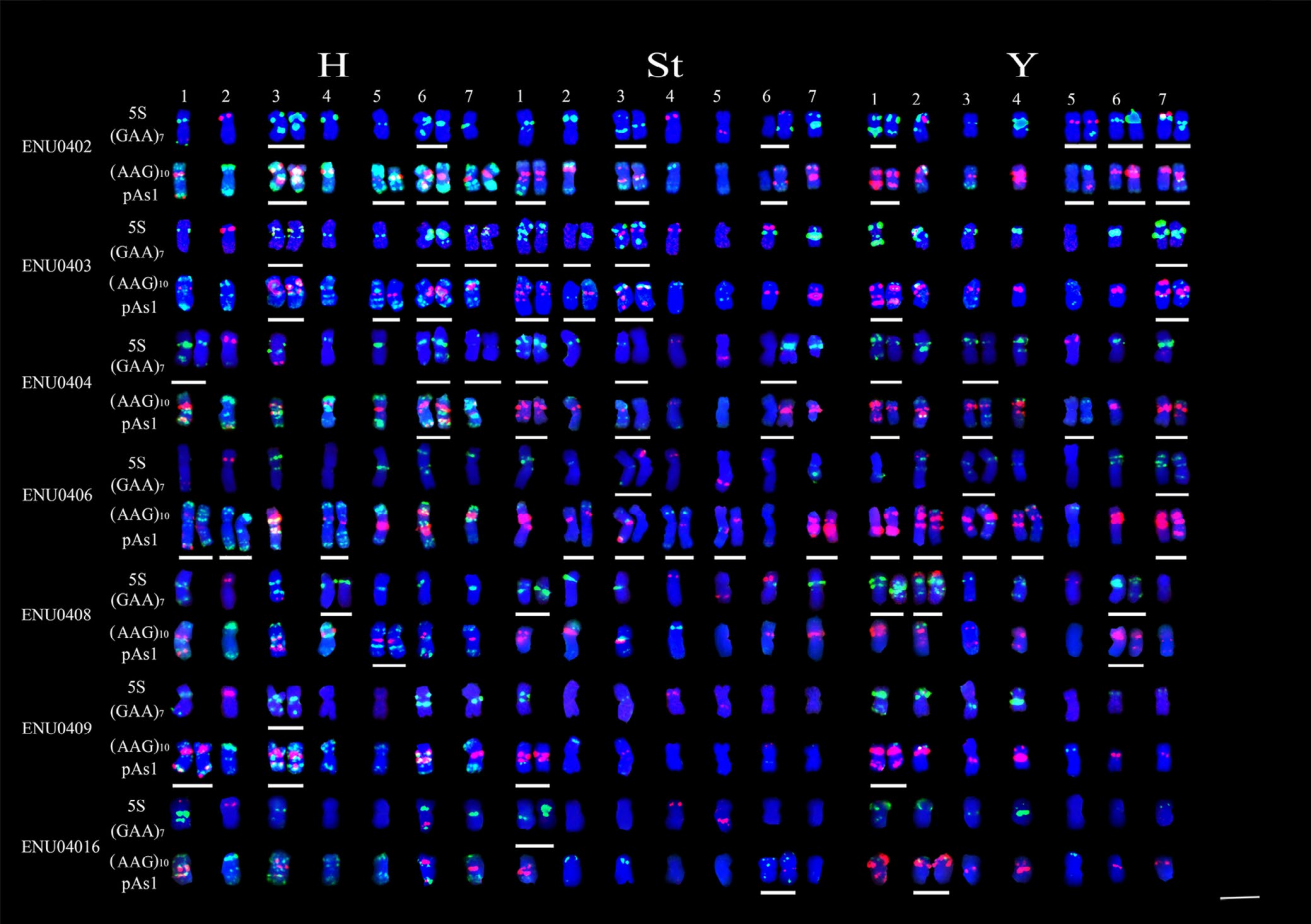
Molecular karyotypic analysis of *C. nutans* Annotation: The fluorescence in situ hybridization (FISH) signals were detected using the following oligonucleotide probes: 5S rDNA (red), (GAA)_7_ (green), (AAG)_10_ (red), and pAs1 (green), as explicitly labeled in the figure. The karyotype exhibits a representative single chromosome from each homologous pair derived from seven altitudinally divergent *C. nutans* ecotypes. Chromosomal polymorphisms within individual accessions are demarcated by white horizontal bars adjacent to the respective chromosomal loci. Scale bar = 10 μm.

**FIGURE 3:**
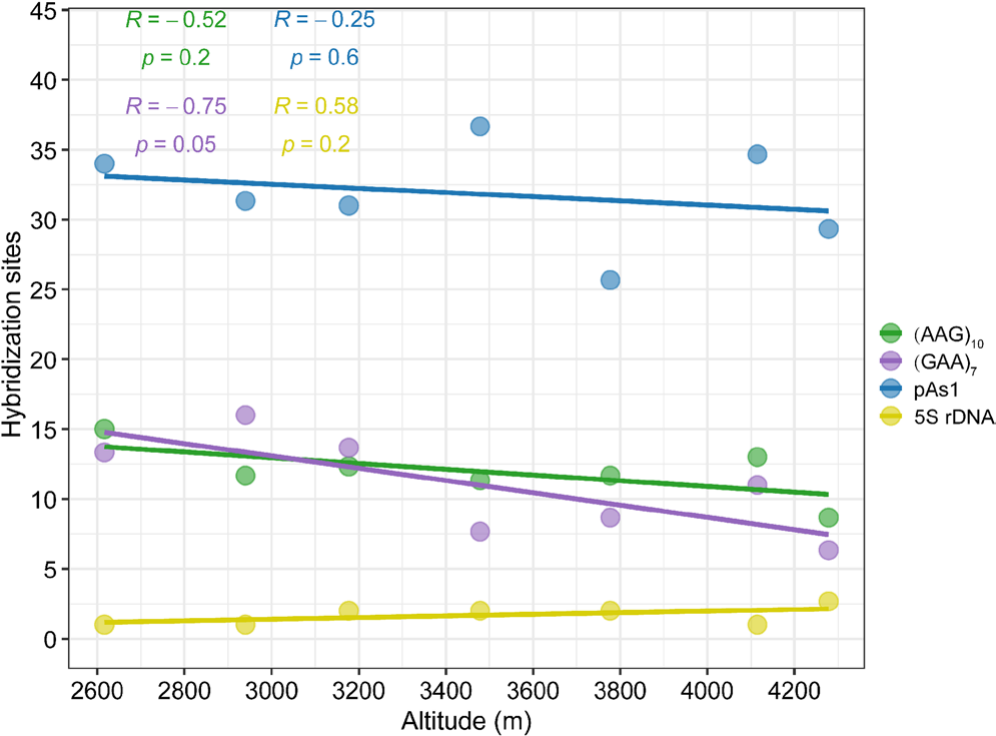
Correlation analysis between hybridization sites and altitude of H genome.

The pAs1 probe predominantly exhibited diffuse signals at chromosomal termini, with minor occurrences at centromeric regions. A subset of signals appeared as discrete loci at both short and long arm termini, while others localized to centromeric positions (Figures 1 and 2). The probe detected signals on all seven chromosome pairs, yielding about 223 hybridization sites with only 14 polymorphic loci, demonstrating high density but low variability (Table 2). Notably, signal abundance exhibited altitudinal variation, with materials ENU0406 (mid‐elevation) and ENU0409 (high‐elevation) showing increased signal density (Figure 3). The (AAG)_10_ probe (red) primarily localized to proximal long arm regions and pericentromeric short arms, displaying partial diffuse patterns near termini. Minor signals appeared as discrete loci at short arm termini and pericentromeric regions. Universal detection across all chromosomes revealed approximately 84 hybridization sites with 12 polymorphic loci, further confirming H genome stability. Signal density peaked in low‐elevation accessions, stabilized in mid‐elevation materials (11–12 sites), and increased again in high‐elevation material ENU0409 (13 sites). The (GAA)_7_ probe (green) showed similar distribution patterns to (AAG)_10_ but was absent on chromosome 2H, detecting signals on only six chromosome pairs. Among a total of approximately 77 hybridization sites, 10 exhibited polymorphism. Signal density fluctuated in low‐elevation accessions but demonstrated an overall decreasing trend with increasing altitude (Figure 3). The 5S rDNA probe (red) primarily localized to the proximal short arm of chromosome 2H, with secondary sites at long arm termini (accessions ENU0404 (3H), ENU0406 (1H), ENU0408 (3H)) and proximal long arm regions (ENU0416 (6H)). Detection on only four chromosome pairs yielded 12 conserved hybridization sites that increased in abundance with elevation. Collectively, these findings demonstrate remarkable structural stability and low polymorphism in the H genome chromosomes of *C. nutans* across altitudinal gradients. Heterozygosity was revealed by the divergent probe hybridization patterns detected between homologous chromosomes (Figure 2). The repetitive sequence distribution patterns exhibit both conserved features and altitude‐dependent variations, suggesting potential adaptive significance in genomic organization.

**TABLE 2:** Number of hybridization sites and chromosomal distribution of repetitive sequences in seven *C. nutans* samples.

	Code	pAs1	(AAG)_10_	(GAA)_7_	5S rDNA
H genome	ENU0402	34 ± 1^b^ (1″2″3″*4″5″*6″*7″*)	15 ± 1^a^ (1″3″*4″5″*6″*7″*)	13.333 ± 0.577^b^ (1″3″*4″5″6″*7″)	1^c^ (2″)
	ENU0403	31.333 ± 0.577^c^ (1″2″3″*4″5″*6″*7″)	11.667 ± 1.155^b^ (1″3″*5″*6″*7″)	16 ± 1^a^ (1″3″*4″5″6″*7″*)	1^c^ (2″)
	ENU0404	31 ± 1^c^ (1″2″3″4″5″6″*7″)	12.333 ± 1.528^b^ (1″2″3″4″5″6″*7″)	13.667 ± 0.577^b^ (1″*3″4″5″6″*7″*)	2^b^ (2″3″)
	ENU0406	36.667 ± 1.528^a^ (1″*2″*3″4″*5″6″7″)	11.333 ± 0.577^b^ (1″*3″4″*5″6″7″)	7.667 ± 1.155^d^ (3″5″6″7″)	2^b^ (1″2″)
	ENU0408	25.667 ± 1.155^d^ (1″2″3″4″5″*6″7″)	11.667 ± 1.155^b^ (1″3″4″5″*6″7″)	8.667 ± 0.577^d^ (1″3″4″*5″6″7″)	2^b^ (2″3″)
	ENU0409	34.667 ± 1.528^b^ (1″*2″3″*4″5″6″7″)	13 ± 1^b^ (1″*3″*5″)	11 ± 1^c^ (1″3″*6″7″)	1^c^ (2″)
	ENU0416	29.333 ± 0.577^c^ (1″2″3″4″5″6″7″)	8.667 ± 1.155^c^ (1″3″6″7″)	6.333 ± 0.577^d^ (1″3″6″7″)	2.667 ± 0.577^a^ (2″6″)
Total hybridization sites		222.67 ± 6.66 (1″*2″*3″*4″*5″*6″*7″*)	83.667 ± 3.786 (1″*2″3″*4″*5″*6″*7″*)	76.667 ± 2.082 (1″*3″*4″*5″6″*7″*)	11.667 ± 0.577 (1″2″3″6″)
Total polymorphic sites		14 (1″, 2; 2″, 1; 3″, 3; 4″, 1; 5″, 3; 6″, 3; 7″, 1)	12 (1″, 2; 3″, 3; 4″, 1; 5″, 2; 6″, 3; 7″, 1)	10 (1″,1; 3″, 3; 4″,1; 6″, 3; 7″, 2)	0
St genome	ENU0402	15.667 ± 1.528^a^ (1″*2″3″*4″5″6″*)	13 ± 1^a^ (1″*2″3″*6″*7″)	10.333 ± 0.577^b^ (1″2″3″*6″*7″)	3^b^ (4″5″6″*)
	ENU0403	13.667 ± 1.528^b^ (1″*2″*3″*4″5″)	13.667 ± 1.155^a^ (1″*2″*3″*6″7″)	13.667 ± 1.155^a^ (1″*2″*3″*6″7″)	3.667 ± 0.577^a^ (3″*4″6″)
	ENU0404	7.667 ± 1.155^c^ (2″3″*4″5″)	11.667 ± 0.577^b^ (1″*2″3″*4″6″*7″)	11.333 ± 0.577^b^ (1″*2″3″*6″*7″)	2^c^ (4″5″)
	ENU0406	7.667 ± 0.577^c^ (1″2″*3″*4″*5″*)	13.667 ± 1.155^a^ (1″2″*3″*4″*5″*7″)	8.333 ± 1.155^c^ (1″3″7″)	4^a^ (3″*4″5″6″)
	ENU0408	6.667 ± 1.155^cd^ (2″4″5″6″)	5.333 ± 0.577^c^ (1″2″3″7″)	9.333 ± 0.577^c^ (1″*2″6″7″)	3^b^ (4″5″6″)
	ENU0409	5.333 ± 0.577^d^ (1″2″5″6″)	7 ± 1^c^ (1″*6″)	3.333 ± 0.577^d^ (1″*3″)	2^c^ (4″5″)
	ENU0416	7 ± 1^cd^ (1″2″3″5″*6″)	3.333 ± 0.577^d^ (1″6″*)	3.667 ± 1.155^d^ (1″*)	2^c^ (4″5″)
Total hybridization sites		63.667 ± 4.163 (1″*2″*3″*4″*5″*6″*)	67.667 ± 5.033 (1″*2″*3″*4″*5″*6″*7″)	60 ± 3 (1″*2″*3″*6″*7″)	19.667 ± 0.577 (3″*4″5″6″*)
Total polymorphic sites		12 (1″, 2; 2″, 2; 3″, 4; 4″,1; 5″, 2; 6″,1)	15 (1″, 4; 2″, 2; 3″, 4; 4″,1; 5″,1; 6″, 3)	10 (1″, 4; 2″,1; 3″, 3; 6″, 2)	3 (3″, 2; 6″, 1)
Y genome	ENU0402	8.667 ± 0.577^c^ (2″3″5″*6″*7″*)	16.667 ± 0.577^b^ (1″*2″3″4″5″*6″*7″*)	16.333 ± 0.577^b^ (1″*2″3″4″5″*6″*7″*)	4^a^ (2″5″*7″*)
	ENU0403	11.333 ± 1.528^a^ (1″*2″3″5″7″*)	15 ± 1^b^ (1″*2″3″4″6″7″*)	15^b^ (1″2″3″4″6″7″*)	1^c^ (2″)
	ENU0404	10^b^ (2″*3″*4″5″*7″*)	16.333 ± 1.528^b^ (1″*2″3″*4″5″6″7″*)	13.667 ± 1.155^b^ (1″*2″3″*4″6″7″)	2^b^ (5″7″)
	ENU0406	7.667 ± 0.577^c^ (2″*3″*4″*5″)	20.333 ± 0.577^a^ (1″*2″*3″*4″*5″6″7″*)	12.667 ± 0.577^b^ (1″2″3″*6″7″*)	1^c^ (2″)
	ENU0408	2.333 ± 0.577^d^ (2″)	13.667 ± 1.528^c^ (1″2″3″4″6″*7″)	18.333 ± 1.155^a^ (1″*2″*3″4″6″*7″)	3.667 ± 0.577^a^ (2″*3″5″)
	ENU0409	4^d^ (1″*2″5″)	15.333 ± 1.155^b^ (1″*2″3″4″6″7″)	10.333 ± 0.577^c^ (1″2″3″4″6″7″)	1^c^ (2″)
	ENU0416	3.333 ± 0.577^d^ (1″2″*5″)	12^c^ (1″2″*3″4″6″7″)	10.667 ± 1.155^c^ (1″2″3″4″7″)	0^d^
Total hybridization sites		47.333 ± 2.309 (1″*2″*3″*4″*5″*6″*7″*)	109.333 ± 0.577 (1″*2″*3″*4″*5″*6″*7″*)	97 (1″*2″*3″*4″5″*6″*7″*)	12.667 ± 0.577 (2″*3″5″*7″*)
Total polymorphic sites		14 (1″, 2; 2″, 3; 3″, 2; 4″, 1; 5″, 2; 6″, 1; 7″, 3)	17 (1″, 5; 2″, 2; 3″, 2; 4″, 1; 5″, 1; 6″, 2; 7″, 4)	12 (1″, 3; 2″, 1; 3″, 2; 5″,1; 6″, 2; 7″, 3)	3 (2″, 1; 5″, 1; 7″, 1)

*Note:* The number before the parenthesis indicates the number of hybridization signal loci, the number in the parenthesis represents the corresponding specific chromosome (Figure 2), the double prime (″) after the number denotes one pair of homologous chromosomes, and the asterisk (*) indicates that chromosomes exhibit polymorphism between two populations of the same species. For the total polymorphic loci, the number following the chromosome represents the total number of polymorphic loci possessed by that chromosome. Different lowercase superscript letters indicate significant differences at *p* < 0.05.

### 3.2 Distribution of Repetitive Sequences in the St Genome of *C. nutans*

**FIGURE 4:**
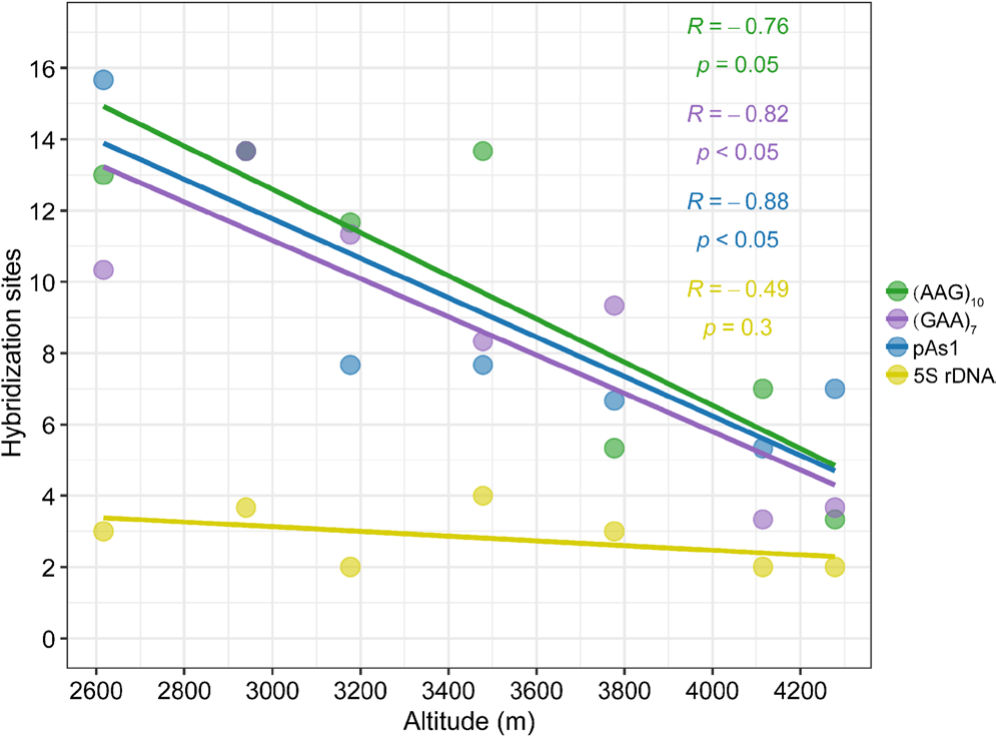
Correlation analysis between hybridization sites and altitude of St genome.

The pAs1 probe hybridization signals were predominantly localized at the terminal regions of chromosome short arms, exhibiting punctate patterns, with some signals diffusely distributed at both termini, albeit faintly (Figures 1 and 2). Hybridization signals were detected on six pairs of chromosomes, with variations observed among materials. For instance, material ENU0409 displayed only about five hybridization sites, whereas ENU0402 exhibited up to about 16 (Table 2). A total of about 64 hybridization sites were identified, including 12 polymorphic sites, with chromosome 3St harboring four polymorphic sites. Notably, the number of signal sites exhibited a declining trend with increasing altitude, particularly at mid‐to‐high elevations (Figure 4). The (AAG)_10_ red probe primarily localized near centromeric regions, displaying diffuse signals, with a minority of punctate signals distributed on the long or short arms adjacent to the centromeres. Signals were detected on all seven pairs of chromosomes, with chromosomes 1St and 3St identified as variation hotspots, each containing four polymorphic sites, while 2St and 6St carried three polymorphic sites, accounting for over half of the polymorphic chromosomes. A total of approximately 68 hybridization sites were observed for (AAG)_10_, including 15 polymorphic sites. The (AAG)_10_ probe exhibited stability at low‐to‐mid altitudes but showed a sharp decline in signal sites at high altitudes (Figure 4). The (GAA)_7_ green probe displayed a similar distribution pattern to (AAG)_10_ but was detected on only five pairs of chromosomes, with no signals observed on 4St or 5St. A total of about 60 hybridization sites were recorded, including 10 polymorphic sites, with chromosome 1St containing four polymorphic sites. The (GAA)_7_ signal sites were more abundant at low‐to‐mid altitudes but relatively reduced at high altitudes. The 5S rDNA red probe exhibited punctate signals with a relatively conserved and stable distribution, primarily localized on chromosomes 4St and 5St. On 4St, signals were mainly concentrated near the terminal region of the short arm, albeit faint in some cases, while on 5St, they were predominantly distributed near the terminal region of the long arm. The remaining 5S rDNA signals were located on the short arms but displayed positional variations. For example, in material ENU0403 (3St), signals were detected near the centromeric region of the short arm, whereas in ENU0404 (3St), they were closer to the short arm terminus. Additionally, materials ENU0402, ENU0403, and ENU0408 exhibited signals at the terminal region of the short arm of 6St. Signals were detected on only four pairs of chromosomes (3St, 4St, 5St, 6St), with a total of approximately 20 sites, including three polymorphic sites. Similar to other probes, 5S rDNA signals were more abundant at low‐to‐mid altitudes. Based on the hybridization signal distribution, the St genome of *C. nutans* exhibits moderate variation and relatively high polymorphism, with the polymorphism gradually decreasing as altitude increased.

### 3.3 Distribution of Repetitive Sequences in the Y Genome of *C. nutans*

**FIGURE 5:**
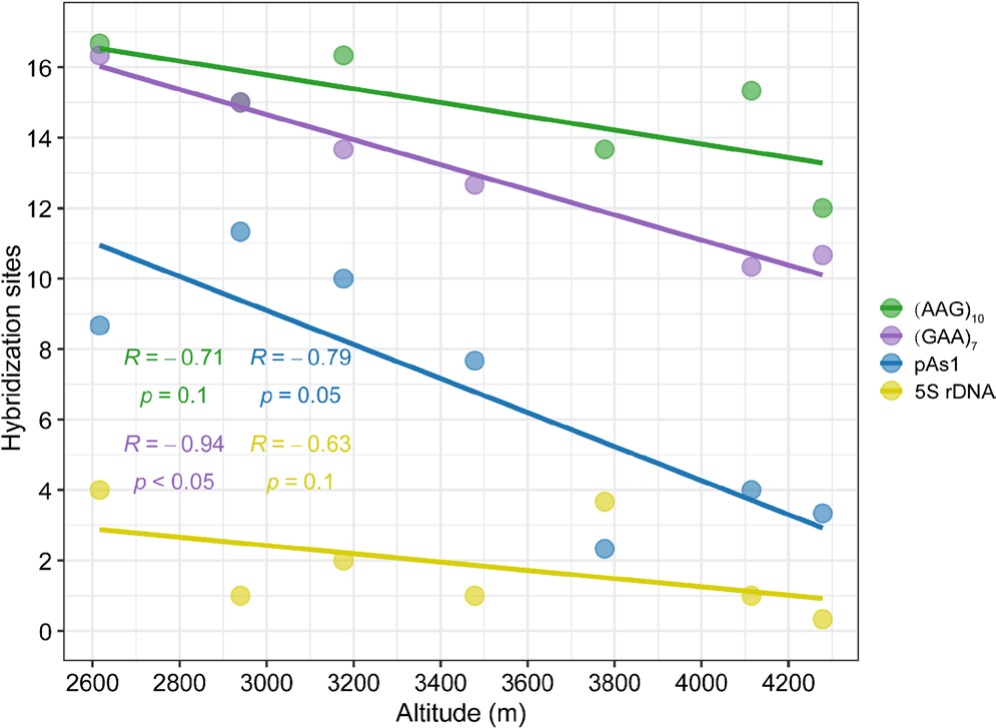
Correlation analysis between hybridization sites and altitude of Y genome.

The hybridization signals of the pAs1 probe were relatively sparse, predominantly appearing as punctate signals at the terminal regions of short arms, with a few localized near centromeres and a minority diffusely distributed at the terminal regions of long arms (Figures 1 and 2). Signals were detected on all seven pairs of chromosomes, with a total of about 47 hybridization sites, including 14 polymorphic sites (Table 2). Chromosomes 2Y and 7Y each contained three polymorphic sites, accounting for half of the total polymorphic chromosomes. The number of signal sites exhibited a slight increase during the transition from low to mid‐altitude regions but sharply declined at higher elevations (Figure 5). The (AAG)_10_ probe displayed a complex signal distribution, primarily appearing as diffuse signals at the terminal regions of long arms and near centromeres, while some punctate signals were observed at the subterminal regions of short arms, centromeric regions, and long arm termini. The (AAG)_10_ probe detected dense signals on all seven chromosome pairs, with a total of approximately 109 hybridization sites, including 17 polymorphic sites. Chromosome 1Y harbored five polymorphic sites, while 7Y contained four. The (AAG)_10_ signals initially increased with elevation, peaking at mid‐altitude (maximum of 21 sites), before decreasing at higher altitudes (Figure 5). The (GAA)_7_ green probe exhibited a distribution pattern similar to (AAG)_10_, with signals detected on all seven chromosome pairs. A total of 97 hybridization sites were recorded, including 12 polymorphic sites, with chromosomes 1Y and 7Y each carrying three polymorphic sites. Similar to (AAG)_10_, the (GAA)_7_ signals fluctuated at low altitudes, reached a maximum at mid‐altitudes, and then declined at higher elevations. The 5S rDNA probe yielded relatively few signals, some of which were faint, primarily appearing as punctate signals at or near the terminal regions of short arms. The distribution was relatively conserved, with signals detected on only four chromosome pairs (2Y, 3Y, 5Y, 7Y), totaling about 13 hybridization sites, including three polymorphic sites. Signal sites were more abundant in materials from low to mid‐altitude regions. Comparative analysis of hybridization signals indicates that the Y genome of *C. nutans* exhibits the highest level of variation and polymorphism among the studied genomes, with the polymorphism of materials from middle and low altitudes being higher than that of materials from high altitudes.

### 3.4 Karyotypic Variation Analysis of *C. nutans* Populations Based on Two Oligonucleotide Probe Combinations

**FIGURE 6:**
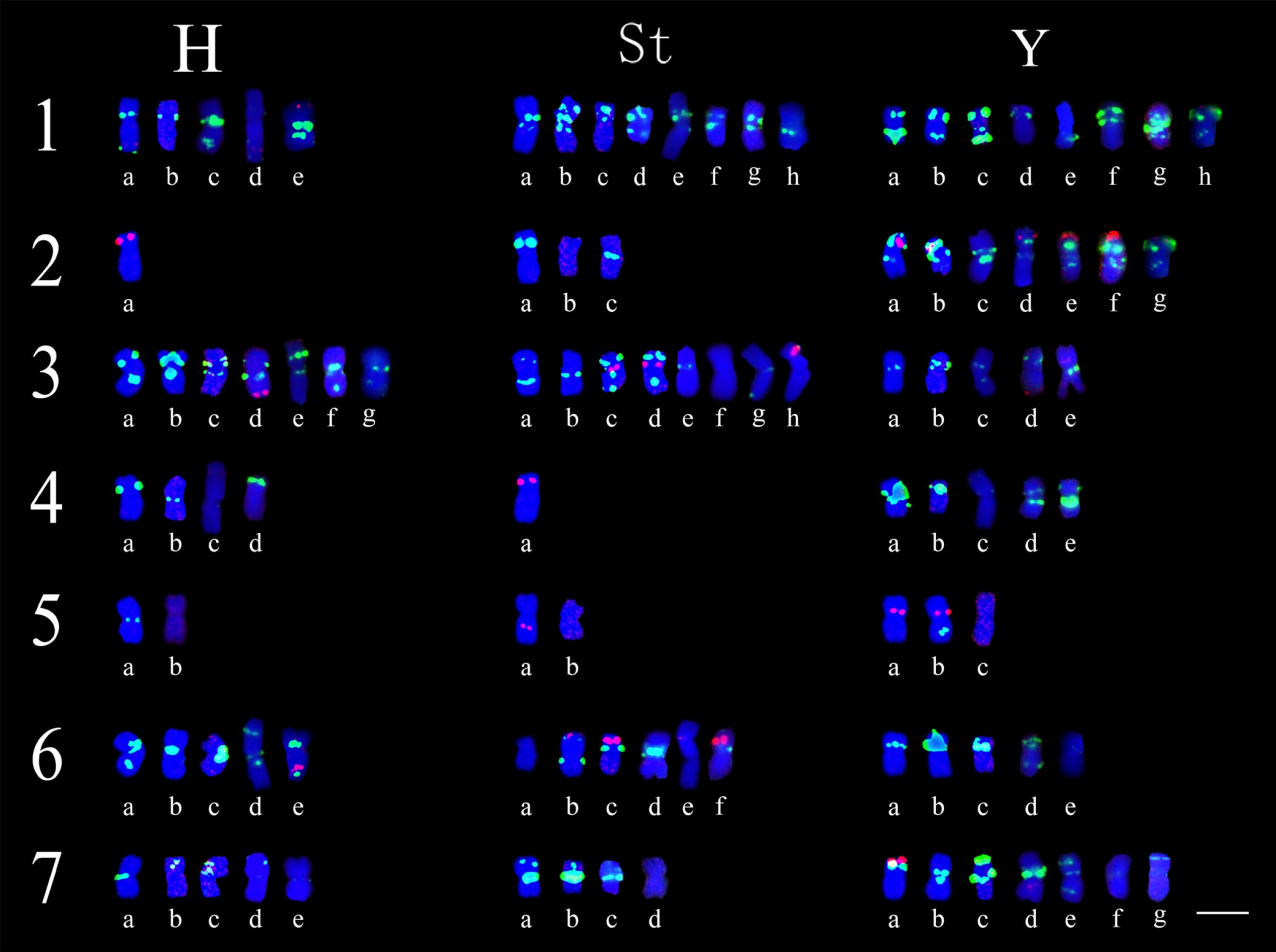
Molecular karyotype analysis of seven *C. nutans* samples from different altitudes based on the 5S rDNA (red) and (GAA)_7_ (green) probe combination.

**FIGURE 7:**
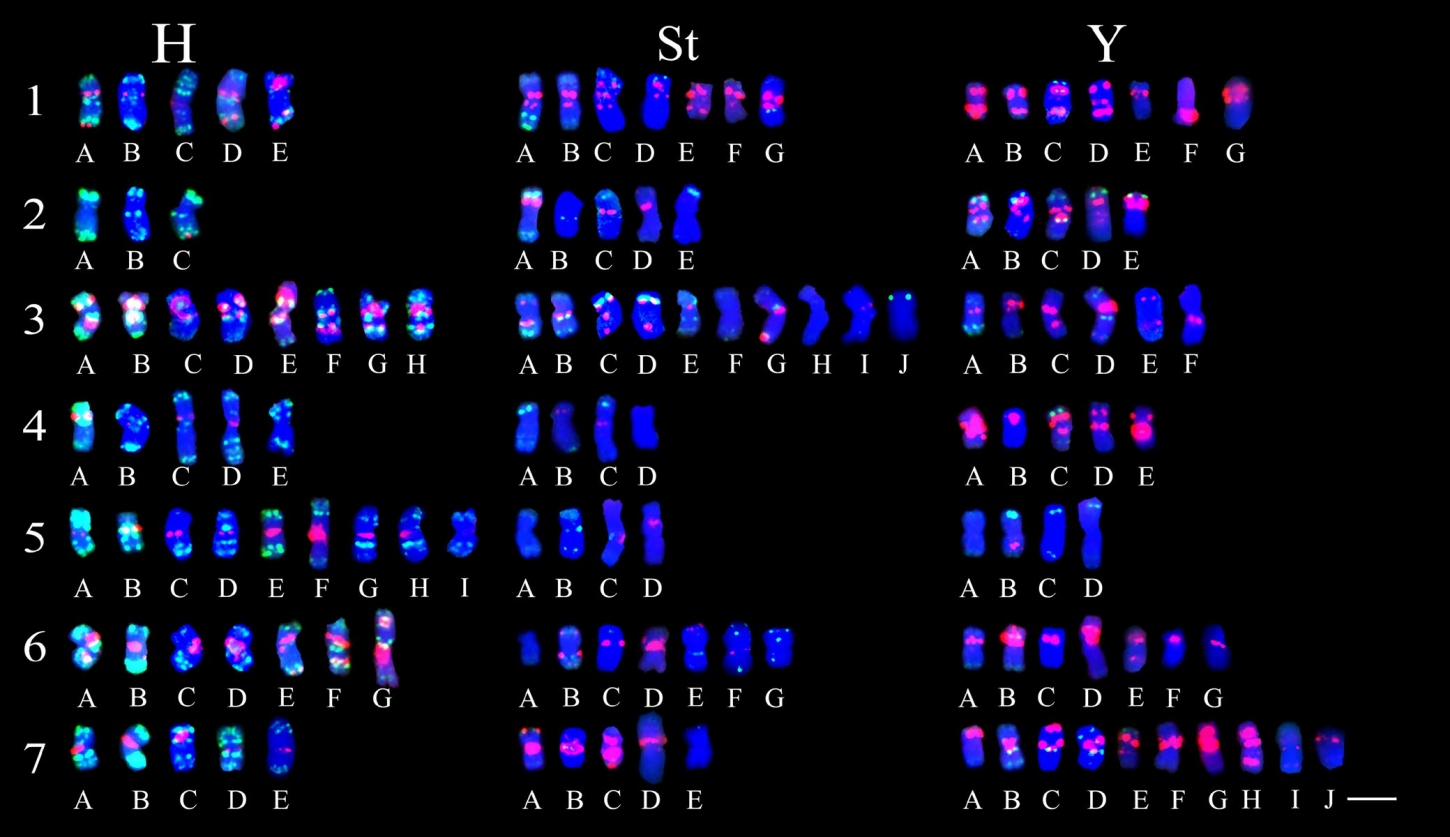
Molecular karyotype analysis of seven *C. nutans* samples from different altitudes based on the (AAG)_10_ (red) and pAs1 (green) probe combination.

Based on the 5S rDNA and (GAA)_7_ probe combination, the results showed that the hybridization signal patterns revealed an uneven distribution of variation types across chromosomes (Figure 6). Within the same genome, different chromosomes (1–7) exhibited varying numbers of variation types, reflecting the preferential occurrence of repetitive sequence amplification/deletion and structural rearrangements. Certain chromosomes were identified as variation “hotspots,” while others remained relatively conserved. The H genome exhibited a total of 29 variation types, with chromosome 3H showing the highest diversity (7 variation types) (Table 3). The St genome displayed 32 variation types, among which chromosomes 1St and 3St showed the highest polymorphism with 8 variation types each. The Y genome exhibited the greatest variation diversity, particularly in chromosomes 1Y (8 types), 2Y (7 types), and 7Y (7 types). Overall, the activity of chromosomal variations marked by 5S rDNA and (GAA)_7_ differed among genomes, with the Y genome being the most variable and the H genome relatively stable. Based on the pAs1 and (AAG)_10_ probe combination, the results indicated an uneven distribution of variation types across chromosomes, with substantial intragenomic differences (Figure 7). Specific chromosomes (e.g., 3St, 7Y) were more susceptible to pAs1 and (AAG)_10_ related repetitive sequence amplification/deletion events, serving as “enrichment zones” for genetic diversity. The H genome displayed 42 variation types across all seven chromosomes, among which 3H (8 types), 5H (9 types), and 6H (7 types) exhibited the highest variation levels (Table 4). The St genome similarly exhibited 42 variation types, with chromosomes 1St (7 types), 3St (10 types), and 6St (7 types) showing particularly prominent polymorphism. The Y genome showed the highest variation diversity with 44 total types, particularly evident in chromosome 7Y which contained 10 variation types. Both probe combinations revealed unevenly distributed chromosomal variation types and distinct variation hotspots across the H, St, and Y genomes, with the Y genome showing the highest diversity and the H genome being relatively conserved.

**TABLE 3:** Molecular karyotype analysis of seven *C. nutans* samples from different altitudes based on the 5S rDNA (red) and (GAA)_7_ (green) probe combination.

Code	Altitude (m)	H genome							St genome							Y genome						
		1	2	3	4	5	6	7	1	2	3	4	5	6	7	1	2	3	4	5	6	7
ENU0402	2616.24	a	a	ab	a	a	ab	a	a	a	ab	a	a	ab	a	ab	a	a	a	ab	ab	ab
ENU0403	2939.92	b	a	bc	b	a	ca	bc	bc	bc	cd	a	b	c	b	c	b	b	b	c	c	cb
ENU0404	3177.01	ca	a	d	a	a	ba	de	da	c	ef	a	a	ad	c	bd	c	ba	a	a	a	d
ENU0406	3478.48	d	a	e	c	a	d	b	e	b	gh	a	a	e	c	e	d	ac	c	c	a	ce
ENU0408	3777.09	a	a	d	ad	a	b	a	fa	a	b	a	a	f	c	fg	ef	d	d	a	bd	f
ENU0409	4114.64	a	a	ef	c	b	a	a	g	b	e	a	a	a	d	b	b	e	e	c	a	g
ENU0416	4278.28	e	a	g	c	b	e	a	hg	b	f	a	a	a	d	h	g	a	e	c	e	g
No. of variants		5	1	7	4	2	5	5	8	3	8	1	2	6	4	8	7	5	5	3	5	7
Total		29							32							40						

*Note:* Taking the signal pattern of each numbered chromosome of material ENU0402 as the reference type, it is denoted as type a. For the chromosomes of other materials under the same genome and the same number, conduct comparison and classification according to the signal patterns: those with signal patterns consistent with the reference type are classified into the same type; when a new signal pattern different from the existing types appears, it is named sequentially as in alphabetical order, which is the variant type of that chromosome number.

**TABLE 4:** Molecular karyotype analysis of seven *C. nutans* samples from different altitudes based on the (AAG)_10_ (red) and pAs1 (green) probe combination.

Code	Altitude (m)	H genome							St genome							Y genome						
		1	2	3	4	5	6	7	1	2	3	4	5	6	7	1	2	3	4	5	6	7
ENU0402	2616.24	A	A	AB	A	AB	AB	AB	AB	A	AB	A	A	AB	A	AB	A	A	A	AB	AB	AB
ENU0403	2939.92	B	B	CD	B	CD	CD	C	CD	BC	CD	A	B	C	B	CD	B	A	B	C	C	CD
ENU0404	3177.01	A	C	B	A	E	EF	D	EF	C	EF	B	A	AD	B	DE	A	BA	C	CA	C	EF
ENU0406	3478.48	AC	AB	E	CD	F	G	C	C	DC	GH	AC	CD	A	C	BF	BC	CD	CD	D	D	GH
ENU0408	3777.09	D	A	F	A	GH	E	E	F	C	B	A	A	C	D	G	B	E	D	A	DE	I
ENU0409	4114.64	DE	B	GH	E	I	F	E	GF	E	I	D	A	E	E	CD	D	F	E	D	F	I
ENU0416	4278.28	D	B	H	E	I	E	E	G	E	J	D	A	FG	E	C	EB	F	E	D	G	J
No. of variants		5	3	8	5	9	7	5	7	5	10	4	4	7	5	7	5	6	5	4	7	10
Total		42							42							44						

*Note:* Taking the signal pattern of each numbered chromosome of material ENU0402 as the reference type, it is denoted as type A. For the chromosomes of other materials under the same genome and the same number, conduct comparison and classification according to the signal patterns: those with signal patterns consistent with the reference type are classified into the same type; when a new signal pattern different from the existing types appears, it is named sequentially as in alphabetical order, which is the variant type of that chromosome number.

## 4 Discussion

### 4.1 The Genetic Diversity of *C. nutans* From Different Populations

Mounting evidence reveals remarkable genetic diversity in *C. nutans* , particularly in Qinghai‐Tibet Plateau populations. Initial studies by Yan et al. (2006) established higher genetic variation in these populations compared to Inner Mongolian counterparts using allozyme and microsatellite markers. Subsequent cytogenetic analyses have substantially expanded our understanding of this diversity. Dou et al. (2009) demonstrated extensive chromosomal polymorphism through FISH‐GISH techniques with AGG satellite repeats and Afa‐family sequences in Qinghai Lake populations (3000–3500 m altitude), revealing differential signal distribution patterns. This chromosomal‐level variation was systematically characterized by Liu et al. (2022a, 2022b), whose molecular karyotyping analysis of fertility‐normal and fertility‐variant *C. nutans* accessions revealed an uneven distribution of chromosomal variations both among genomes and among homologous chromosomes within each genome, with a consistent descending gradient of polymorphism from the Y genome to the St and H genomes. Our molecular karyotyping analysis employing four oligonucleotide probes also demonstrated a progressive decrease in chromosomal polymorphism across the Y, St, and H genomes in *C. nutans* , with 1Y, 2Y, and 7Y identified as variation hotspots. However, for the tetraploid species *Elymus sibiricus* with the StH genomic constitution, signals from the pAs1 and (AAG)_10_ oligonucleotide probes were stronger on the H genome chromosomes and exhibited relatively higher signal polymorphism (Xie et al. 2020). It was speculated that the increased polymorphism of the Y genome might represent an adaptive response to the environment. Future studies could verify this by performing transcriptome or sequencing analyses on the identified chromosomal hotspot regions. pAs1 and pHvG39 probes were used to study the chromosomal polymorphism of *Kengyilia grandiglumis* with the StPY genome composition, and found that the signal variation was the greatest on chromosomes 1Y, 2Y and 3Y (Wang et al. 2010). Whether chromosomes 1Y and 2Y in Triticeae species are generally prone to variation requires more research in the future. The universality of this genomic hierarchy is further supported by population studies. Liu et al. (2024) analyzed seven *C. nutans* accessions from major global distribution sites and revealed substantial variation in the distribution patterns and quantitative characteristics of S5 and AAG probe signals both within and among populations. Their findings demonstrate that *C. nutans* exhibits high genetic diversity at the chromosomal level across intra‐ and inter‐population. Dou et al.'s (2011) study on the molecular karyotype of *C. nutans* from northwestern China revealed fewer 5S rDNA hybridization signal sites in both H and St genomes, while AAG repeats were significantly more abundant in the H and Y genomes compared to the St genome. Dou et al. (2017) employed (AAG)_10_ and pAs1 probes to analyze the chromosomal architecture of *C. nutans* populations from different altitudes in Qinghai, revealing significantly higher polymorphism in the St and Y genomes compared to the H genome. These findings were consistent with the present study results, demonstrating the relatively conserved chromosomal architecture of *C. nutans* , especially in the H subgenome. The uneven variation between genomes and chromosomes—including subgenome dominance, partial repeat sequence elimination, and diploidization processes—represents a widespread phenomenon in the evolution of polyploid plants (Cheng et al. 2018; Session 2024; Li et al. 2021). Through complex genetic and epigenetic mechanisms, these processes collectively drive adaptive evolution, species divergence, and trait innovation in plants, providing abundant genetic resources for crop improvement (Wang et al. 2022; Saul et al. 2023). In this study, we found that signals of (AAG)_10_ and (GAA)_7_ peaked at middle elevations (approximately 3000–4000 m) and decreased at higher elevations. Simple sequence repeats (SSRs) tend to generate extensive length polymorphism owing to strand slippage during DNA replication, and such variation provides abundant genetic resources for plants to achieve rapid adaptation to environmental changes (Sureshkumar et al. 2025). As highly polymorphic regions in the genome, microsatellite repeats can rapidly generate novel genetic variation through expansion or contraction, providing more raw material for natural selection (Li et al. 2002; Gao et al. 2013). The peaks of (AAG)_10_ and (GAA)_7_ at middle elevations may indicate that these repeat sequences assist *C. nutans* in better coping with moderate stresses such as low temperature, drought, or high light in middle‐elevation environments by influencing the expression or regulatory networks of key genes, thereby achieving an optimal adaptive state (Guo et al. 2020). The stress intensity at middle elevations likely falls within the threshold tolerable for genome instability in *C. nutans* . Within this threshold, genome plasticity conferred by the expansion or contraction of microsatellite repeats such as (AAG)_10_ and (GAA)_7_ allows plants to rapidly adjust gene expression and better adapt to fluctuating environments (Ranathunge et al. 2018; Pires et al. 2024). Despite the vital role of dynamic expansion of repetitive elements in plant adaptation to abiotic stress, studies on the Himalayan alpine genus Roscoea and the Qinghai‐Tibet Plateau endemic cruciferous species Crucihimalaya lasiocarpa revealed that their karyotypes have remained highly conserved during long‐term evolution under extreme environmental stress (Feng et al. 2022; Wang et al. 2024). This indicates that alpine plants at high altitudes generally tend to maintain stable karyotypes, which may represent an evolutionary strategy balancing genome plasticity and stability.

Compared with satellite sequences, it was found that 5S rDNA increased with rising elevation. Ribosomal DNA (rDNA) encodes ribosomal RNA (rRNA), an essential component of ribosomes—the molecular machinery responsible for protein synthesis (Hori et al. 2023). The expansion of 5S rDNA in *C. nutans* under high‐altitude conditions may represent an adaptive strategy to meet elevated metabolic demands and enhance responsiveness to diverse environmental stresses (Goffová and Fajkus 2021). Plant genomes are highly plastic, allowing them to adapt to environmental changes through genomic rearrangements, copy number variations, and epigenetic modifications (Zhang et al. 2024; Dinkar et al. 2024). The reduction in pAs1 hybridization sites in high‐altitude *C. nutans* may represent one manifestation of such genomic plasticity under specific environmental selection pressures. Mechanistically, Liu et al. (2022a, 2022b) discovered that chromosomal variations in *C. nutans* originate from both pre‐existing inversions in parental populations and de novo structural changes during meiosis, with secondary rearrangements being the main driver of widespread meiotic abnormalities and genomic diversity. These findings partially elucidate the origins of the remarkable genetic diversity observed in *C. nutans* , a species widely distributed across the Qinghai‐Tibet Plateau. Species‐specific intra‐genomic chromosomal rearrangements were identified in H, St and Y genomes of *C. nutans* using single‐gene fluorescence in situ hybridization (FISH) probes, demonstrating its remarkable intra‐genomic genetic diversity (Liu et al. 2023). For the materials investigated in this study, the detection of finer‐scale intra‐genomic chromosomal variations would require the application of more specific probes. Collectively, these studies demonstrate *C. nutans* ' exceptional chromosomal plasticity, particularly in its Qinghai‐Tibet Plateau adaptation.

### 4.2 Environmental Determinants of Chromosomal Structural Polymorphism in Alpine Grass *C. nutans*

Polyploidy confers significant ecological advantages, enabling broader geographical distribution than diploid ancestors (Liu and Wang 2023), particularly in extreme environments. Studies across diverse taxa demonstrate this pattern. In the South African genus *Schoenus* (Cyperaceae), Elliott et al. (2023) found that polyploid species occurred more frequently in drier regions and areas with more variable climates compared to diploid species. For the genus *Allium*, polyploids showed a stronger tendency to be distributed in higher‐altitude areas with harsher environmental conditions (Wang, Zhou, et al. 2023). FISH signals were largely conserved among geographically distinct *Haynaldia villosa* accessions, but reproducible signal differences were detected between the UK‐derived accession 91C43 and the five US accessions, reflecting chromosomal diversification shaped by geographical differentiation (Lei et al. 2020). Thirteen *Dasypyrum villosum* accessions from diverse geographic regions including Bulgaria, Ukraine, Italy, Turkey, Greece, and the UK were analyzed by multiplex oligonucleotide FISH, which revealed significant chromosomal polymorphisms and population differentiation in repetitive sequence signals, reflecting genome diversification driven by geographical isolation and adaptive evolution (Wu et al. 2022). The Qinghai‐Tibet Plateau exemplifies this phenomenon as an “evolutionary cradle” for angiosperm polyploidization, harboring significantly higher polyploid and paleopolyploid frequencies than eastern regions (Wang, Deng, et al. 2023). This advantage stems from polyploids' enhanced genetic diversity through genome doubling/hybridization (Luttikhuizen et al. 2007), facilitating adaptation to extreme conditions. *C. nutans* exemplifies polyploid adaptation mechanisms. Cold stress is likely a key inducer of cytomixis and associated meiotic abnormalities in the gametic cells of *C. nutans* anthers, ultimately leading to reduced pollen fertility (Singh et al. 2020). At the physiological level, high‐altitude *C. nutans* achieves enhanced chilling tolerance through dynamic coordination of phytohormones and sugars, coupled with efficient expression of EnCBFs/EnCOR genes (Fu et al. 2016). High genetic diversity is beneficial for *C. nutans* to deal with environmental hardness from a genetic perspective (Dou et al. 2009). Chen et al. (2009) conducted an ISSR‐based analysis of 63 *C. nutans* accessions across western China, demonstrating significant genetic differentiation between Qinghai‐Tibet Plateau and Xinjiang populations, with climate, topography and elevation serving as primary drivers of this divergence. Their findings further support the Qinghai‐Tibet Plateau's role as a potential genetic diversity center for this important forage species.

Chen et al. (2013) analyzed 50 *C. nutans* accessions from the eastern Qinghai‐Tibet Plateau using simple sequence repeat (SSR) markers, revealing high genetic diversity and a broad genetic base among the accessions. These findings align with broader patterns of genetic variation observed across altitude gradients in the region. Yan et al. (2009) employed fluorescence‐based AFLP fingerprinting to analyze the hexaploid species *C. nutans* , demonstrating an altitudinal pattern of genetic diversity characterized by an initial increase followed by a subsequent decrease, with peak diversity observed at mid‐altitude ranges. Genomic analysis of *C. nutans* populations reveals altitudinal expansion from mid‐elevation to both higher and lower altitude ranges (Xiong et al. 2025). Notably, the mid‐altitude zones appear particularly significant for genome evolution; larger genome size variations occurred in the mid‐altitude (3900–4300 m) populations of *C. nutans* compared with other‐altitude populations, suggesting a notable altitudinal pattern in genome size variation, which shaped genome evolution by altitude (Chen et al. 2022). The mid‐altitude regions (3000–4000 m) of the Western Sichuan Plateau not only served as the primary distribution area for *C. nutans* but also, as confirmed by the study, represent the zone where the species exhibited the highest genetic diversity. Similar patterns have been observed in other alpine species. The high‐altitude populations of *Potentilla fruticosa* L. on the Qinghai‐Tibet Plateau not only maintain higher genetic diversity but also preserve ancestral haplotypes, whereas their low‐altitude counterparts exhibit reduced genetic diversity with exclusively recently derived haplotypes (Shimono et al. 2010). Based on sampling across seven elevation gradients, this study found that mid‐altitude populations exhibit higher chromosomal genetic diversity, suggesting that the mid‐altitude region of the Western Sichuan Plateau is the genetic diversity center of *C. nutans* and may deserve priority conservation. Reproductive adaptations complement these genetic strategies. To adapt to high‐altitude habitats with pollinator scarcity, many alpine plants have evolved self‐compatible breeding systems through facultative self‐pollination from obligately outcrossing low‐altitude ancestors (Liu et al. 2014). As a perennial polyploid forage grass, *C. nutans* predominantly exhibits cross‐pollination, though its high‐altitude populations showed reduced genetic diversity likely due to increased self‐pollination under extreme alpine conditions. These multilayered adaptations—from chromosomal to physiological and reproductive levels—underscore *C. nutans* ' successful colonization of the Plateau's variable altitudes.

## Author Contributions

**Cairong Yang:** conceptualization (equal), data curation (equal), project administration (equal), supervision (equal), writing – original draft (equal), writing – review and editing (equal). **Jiezhi Yang:** formal analysis (equal), investigation (equal), methodology (equal), writing – original draft (equal). **Yi Ou:** data curation (equal), formal analysis (equal), investigation (equal), methodology (equal). **Yueju Zhou:** data curation (equal), formal analysis (equal), investigation (equal), methodology (equal). **Zhimeng Wang:** formal analysis (equal), investigation (equal), methodology (equal). **Weiliang Qi:** visualization (equal), writing – review and editing (equal). **Dingfang Luo:** investigation (equal), methodology (equal), visualization (equal). **Yutong Qiao:** investigation (equal), methodology (equal), visualization (equal).

## Conflicts of Interest

The authors declare no conflicts of interest.

## Data Availability

All data supporting the findings of this study are included within the manuscript.
